# New Perspectives for Eye-Sparing Treatment Strategies in Primary Uveal Melanoma

**DOI:** 10.3390/cancers14010134

**Published:** 2021-12-28

**Authors:** Krzysztof Bilmin, Kamil J. Synoradzki, Anna M. Czarnecka, Mateusz J. Spałek, Tamara Kujawska, Małgorzata Solnik, Piotr Merks, Mario Damiano Toro, Robert Rejdak, Michał Fiedorowicz

**Affiliations:** 1 Department of Health Sciences, Jan Długosz University in Częstochowa, 42-200 Częstochowa, Poland; kbilmin@wp.pl; 2 Small Animal Magnetic Resonance Imaging Laboratory, Mossakowski Medical Research Institute, Polish Academy of Sciences, 02-106 Warsaw, Poland; mfiedorowicz@imdik.pan.pl; 3 Department of Experimental Pharmacology, Mossakowski Medical Research Institute, Polish Academy of Sciences, 02-106 Warsaw, Poland; anna.czarnecka@gmail.com; 4 Department of Soft Tissue/Bone Sarcoma and Melanoma, Maria Sklodowska-Curie National Research Institute of Oncology, 02-781 Warsaw, Poland; mateusz@spalek.co; 5 Institute of Fundamental Technological Research, Polish Academy of Sciences, 02-106 Warsaw, Poland; tkujaw@ippt.pan.pl; 6 Faculty of Medicine, Medical University of Warsaw, 02-091 Warsaw, Poland; m.solnik98@gmail.com; 7 Department of Pharmacology and Clinical Pharmacology, Faculty of Medicine, Collegium Medicum Cardinal Stefan Wyszyński University, 01-815 Warsaw, Poland; p.merks@uksw.edu.pl; 8 Department of Ophthalmology, University of Zurich, 8091 Zurich, Switzerland; toro.mario@email.it; 9 Chair and Department of General and Pediatric Ophthalmology, Medical University of Lublin, 20-079 Lublin, Poland; robertrejdak@yahoo.com

**Keywords:** uveal melanoma, HIFU, iontophoresis, electrotherapy, nanoparticles, theranostics

## Abstract

**Simple Summary:**

Uveal melanoma is the most common intraocular cancer. The current eye-sparing treatment options include mostly plaque brachytherapy. However, the effectiveness of these methods is still unsatisfactory. In this article, we review several possible new treatment options. These methods may be based on the physical destruction of the cancerous cells by applying ultrasounds. Another approach may be based on improving the penetration of the anti-cancer agents. It seems that the most promising technologies from this group are based on enhancing drug delivery by applying electric current. Finally, new advanced nanoparticles are developed to combine diagnostic imaging and therapy (i.e., theranostics). However, these methods are mostly at an early stage of development. More advanced studies on experimental animals and clinical trials would be needed to introduce some of these techniques to routine clinical practice.

**Abstract:**

Uveal melanoma is the most common intraocular malignancy and arises from melanocytes in the choroid, ciliary body, or iris. The current eye-sparing treatment options include surgical treatment, plaque brachytherapy, proton beam radiotherapy, stereotactic photon radiotherapy, or photodynamic therapy. However, the efficacy of these methods is still unsatisfactory. This article reviews several possible new treatment options and their potential advantages in treating localized uveal melanoma. These methods may be based on the physical destruction of the cancerous cells by applying ultrasounds. Two examples of such an approach are High-Intensity Focused Ultrasound (HIFU)—a promising technology of thermal destruction of solid tumors located deep under the skin and sonodynamic therapy (SDT) that induces reactive oxygen species. Another approach may be based on improving the penetration of anti-cancer agents into UM cells. The most promising technologies from this group are based on enhancing drug delivery by applying electric current. One such approach is called transcorneal iontophoresis and has already been shown to increase the local concentration of several different therapeutics. Another technique, electrically enhanced chemotherapy, may promote drug delivery from the intercellular space to cells. Finally, new advanced nanoparticles are developed to combine diagnostic imaging and therapy (i.e., theranostics). However, these methods are mostly at an early stage of development. More advanced and targeted preclinical studies and clinical trials would be needed to introduce some of these techniques to routine clinical practice.

## 1. Introduction

**Figure 1:**
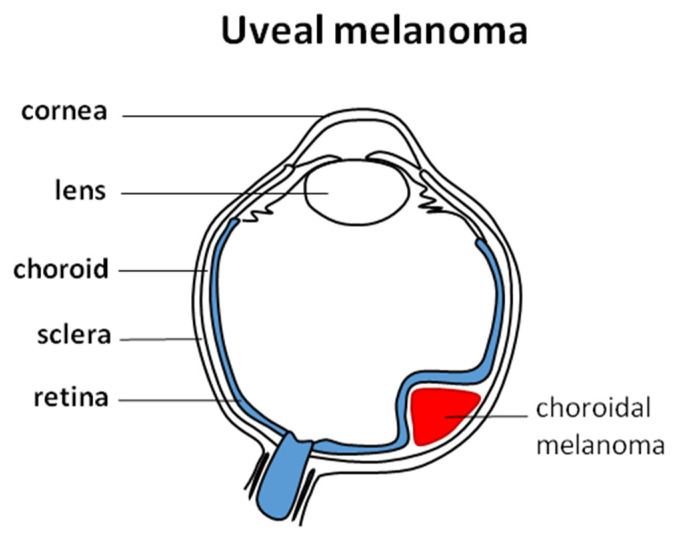
Uveal melanoma and its typical localization in the choroid.

Uveal melanoma (UM) is the most common intraocular malignancy and arises from melanocytes in the choroid (90%, Figure 1), ciliary body (6%), or iris (4%) [1]. In UM, the median age at diagnosis is 62 years; congenital or infantile melanoma is rare and is not common in children [2,3]. It is a disease with a poor prognosis as in the metastatic setting; expected overall survival is no longer than 12 months [4]. In Europe, UM incidence increases with latitude and ranges from 2/106 in Spain and Italy, 4–6/106 in Central Europe, and >8/106 in Denmark and Norway. Worldwide UM incidence is 1–9/100,000 [5]. Around 50% of patients with UM will develop metastasis regardless treatment with average survival time of 6–12 months [6,7].

UM’s signs and symptoms are non-specific and include high intraocular pressure, myodesopsia, photopsia, and, finally, loss of vision. These tumors are most often detected incidentally in an ophthalmological exam [8]. Besides fair skin type and sunlight ultraviolet (UVA/UVB) exposures, general risk factors of UM development, mutations in the tumor suppressor gene, encoding BRCA1 associated protein 1 (BAP1) have been found to increase the risk of UM development. *BAP1* mutations are detected in 47% of cases [9] Furthermore, *GNAQ* and *GNA11* gene mutations are often detected in UM and *SF3B1* and *EIF1AX* gene mutations. Moreover, mutations in the promoter of *BRAF*, *NRAS*, and *TERT* genes have been described. The presence of *TETR*, *BAP1*, *SF3B1*, and *EIF1AX* mutations has a prognostic significance [10].

The management of localized UM is either globe-preserving therapy or enucleation. Globe-preserving therapies may be surgical, radiation therapy, or laser therapy. In general, all the treatments are still unsatisfactory in terms of local disease control, as the average treatment failure in all radiation therapies is 6.15%, 18.6% in surgical, and 20.8% in laser therapies [11]. In particular, radiation therapy modalities include brachytherapy, photon-based external-beam radiation, and charged-particle radiation. For brachytherapy reported local recurrence rates are 14.7% for ^106^Ru treatment, 7–10% for ^125^I, and 3.3% for ^103^Pd. Brachytherapy does not lead to increased survival rates as compared to enucleation [5].

On the other hand, photodynamic laser photocoagulation and transpupillary thermal therapy (TTT) are treatment modalities that directly focus energy to destroy tumor vasculature and reduce local recurrences by injecting and activating light-sensitive compounds and free radicals. TTT was effective in 80% of cases of small or indeterminate lesions with few risk factors. Finally, no adjuvant (chemo) therapy has been shown to prolong survival to date [5].

This study aimed to review current and prospective approaches in the eye-preserving treatment of localized ocular melanoma.

## 2. Ocular Pharmacology

Ocular pharmacology is unique. The drug administration routes and therapeutic challenges vary depending on the eye segment [12]. The anterior segment of the eye includes the cornea, iris, ciliary body, and lens. Topical application (e.g., drops) is the most common form of pharmacotherapy in this segment. Since the volume of an eye drop (50 μL) significantly exceeds the normal tear volume (7 μL), most drugs get drained by the nasolacrimal duct or spill over the eyelids.

Moreover, irritant drugs may induce lacrimation causing drug loss. Other factors affecting topical drug residence time include tear film turnover and low corneal permeability [12,13]. This results in low bioavailability (<5%) of topically applicated treatment [14]. Additionally, self-administration of drops requires high patient compliance, which impacts the therapeutic efficacy. To improve drug contact time and result efficiency in drug delivery, various strategies have been utilized. This includes the development of mucoadhesive polymeric gels, ointments, liposome formulations to increase the carrier’s viscosity, and the introduction of sustained and controlled-release therapeutics, such as hydrogel lenses or collagen shields, or drug-cyclodextrin complexes, which increase the aqueous solubility of drugs [13,15,16].

The tissues of the posterior segment of the eye (vitreous humor, retina, choroid, optic nerve, sclera) are mostly reached by systemic and intraocular drug administration [13]. However, oral or intravenous routes are heavily impaired due to the presence of ocular blood barriers. The blood–aqueous barrier (BAB) consists of the non-pigmented epithelium of the ciliary body, the endothelial cells in the iris vessels, the posterior iridial epithelium, and the Schlemm canal endothelium. On the other hand, the blood–retinal barrier (BRB) is formed by the retinal pigment epithelial cell layer and retinal vascular endothelium [15,16]. Both are responsible for maintaining intraocular homeostasis and restricting the passage of blood elements and macromolecules into eye chambers [13,15]. Consequently, limited drug influx into the retina and vitreous body require administering high doses of systemic drugs, which causes various undesirable side effects [17]. With recent developments in nanomedicine, the use of nanoparticles in systemic drug delivery may overcome ocular physiological barriers since they have shown the ability to pass through capillaries and reach the desired site with high selectivity via specific targeting systems [15]. Hence, intravitreal injections remain the most straightforward method in drug administration to the posterior eye segment. However, they mostly require repeated injections, resulting in potential side effects including retinal detachment, intraocular hemorrhage, infection, and endophthalmitis [13,18]. Additionally, the procedure is unpleasant for the patient and must be performed by an ophthalmologist, which requires monthly or bi-monthly visits [12]. Intravitreal sustained-release devices have been introduced to address the issue (e.g., Vitrasert ganciclovir implant for cytomegalovirus retinitis treatment). The devices are inserted through intraocular surgery; they require periodic replacements, and potential complications are similar to intravitreal injections [17].

## 3. Radiotherapy

**Figure 2:**
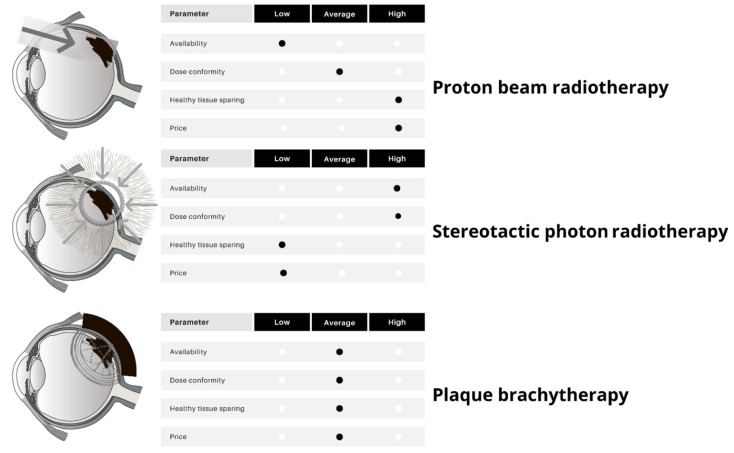
Radiotherapy techniques for uveal melanoma: characteristics, main advantages, and disadvantages.

Radiotherapy (RT) is the mainstay of therapy for most patients with localized uveal melanoma. Due to the predicted radioresistance of melanoma cells, high fraction doses are required to achieve a satisfactory response and local control [19]. Thus, the preferred RT methods comprise plaque brachytherapy (BT), charged-particle RT (CRT), and photon-based stereotactic RT (SBRT) [20,21]. Conventional photon-based external beam radiotherapy is not recommended due to no benefit in survival [22]. Importantly, the occurrence of local relapse of uveal melanoma is related to the risk of distant metastases [23,24]. In a systematic review of 49 studies on local therapies for uveal melanoma, the authors reported the local treatment failure rate from 0% to 55.6%, with follow-up ranging from 10 to 150 months. The weighted average of local failure in all RTs was 6.15% compared with 18.6% in eye-sparing surgeries and 20.8% in laser therapies. However, local control rates varied even between centers that used similar techniques. Therefore, proper RT modality is crucial and should be based on various factors and institutional experience (Figure 2 and Table 1).

### 3.1. Brachytherapy

Plaque BT is the most widespread RT method for uveal melanoma available in many specialized RT departments. The main principle is to place radioactive isotopes on the outer surface of the sclera and deliver the prescribed dose to the target volume. It requires ophthalmological surgery due to plaque insertion, suturing, and plaque removal. The irradiation usually lasts between two and four days [25].

Plaque BT replaced enucleation due to favorable results of the COMS study meta-analysis published in 2006 that showed equivalence in the survival of patients with choroidal melanomas randomly allocated to receive iodine-125 brachytherapy or enucleation [26]. The used isotopes include cobalt-60, iodine-125 (^125^I), iridium-192, palladium-103, and ruthenium-106 (^106^Ru). In clinical practice, the most frequently applied isotopes are ^106^Ru and ^125^I due to their wide availability and favorable dose distribution. However, ^125^I is preferred in larger tumors due to its physical properties, namely, emission of gamma radiation which penetrates deeper than beta-emitters like ^106^Ru. These observations were confirmed in several studies [27,28,29,30,31]. Intraoperative ultrasonography to verify plaque placement improved the treatment results, especially for tumors localized in anatomically challenging eye parts, such as its posterior area [32,33,34].

According to the consensus opinion guidelines published by the American Brachytherapy Society, most melanomas of the iris, ciliary body, and choroid could be treated with BT. Data regarding application technique, dose rate, dosimetry, and quality assurance are presented in detail in this consensus [35].

### 3.2. Stereotactic Body Radiotherapy

Photon-based SBRT is also a viable treatment option for uveal melanoma; however, it has been less investigated than BT and CRT. The indisputable advantage of SBRT is the broad accessibility to this technique based on linear accelerators available in the majority of RT departments. The most common fractionation regimen comprises between 50 and 70 Gy given in five successive fractions. Despite much less extensive experience with SBRT for uveal melanoma than for other RT methods, data regarding local efficacy seem to be equivalent. However, SBRT is linked with a higher risk of late complications than BT and PT [36].

### 3.3. Charged-Particle Radiotherapy

Another RT technique used in the treatment of uveal melanomas is CRT. It includes protons, carbon ions, and helium ions [37,38,39,40]. Unfortunately, the availability of CRT, especially ion therapy, remains poor due to the high cost of equipment and treatment (availability according to the data by the Particle Therapy Co-Operative Group [41]). The unique physical properties of CRT beams, namely, the Bragg peak, allow the deposition of most energy in a precisely defined volume with subsequent sharp dose fall-off behind the target volume. CRT provides excellent local control around 90%, similar to that achieved with BT [42]. Nonetheless, due to external beam delivery, there is still the risk of damage to eye structures such as lashes, macula, retina, lens, iris, or cornea.

Interestingly, worse local control after CRT could be associated with several factors: reduced safety margins, presence of large ciliary body tumors, presence of eyelids within the treatment field, wrong positioning of tantalum clips, and male gender [43].

### 3.4. Ocular Complications of RT

The risk of ocular complications after RT depends on many factors, including the technique, delivered dose, margins, tumor size, and comorbidities. The ocular complications comprise retinopathy, cataracts, maculopathy, vitreous hemorrhage, retinal detachment, strabismus, secondary glaucoma, optic neuropathy, scleral necrosis, uveitis, and others [44].

Brachytherapy is the most common treatment option for patients with small- and medium-sized UM, allowing preservation of the eye globe. However, this treatment is associated with possible severe adverse reactions. The most frequent complications include radiation-induced retinopathy (45–67%), cataracts (44%), neovascular glaucoma (28%), and macular edema (25%). They can result in moderate vision loss in 58% of patients and poor visual acuity (best corrected worse than 5/200) in 28% within two years [5]. Therefore, the development of safer treatment modalities is needed. 

The frequent ocular complication of RT is cataracts. The risk factors include the total dose (especially over 12 Gy) and anterior tumors (65–90% risk of cataract development). The most efficient management is cataract surgery that could be safely performed despite previous irradiation.

Retinopathy may manifest clinically or be asymptomatic. Early diagnosis of radiation-induced retinopathy can be performed using optical coherence tomography. The typical signs are telangiectasia, exudates, cotton wool spots, and microaneurysms. In advanced stages, it could lead to vision loss due to ischemic necrosis. The most important risk factors of radiation-induced retinopathy are diabetes, hypertension, total delivered dose, thick tumors, and proximity of the target volume to the foveola. The available treatment methods include photodynamic therapy, laser photocoagulation, vitrectomy, oral pentoxifylline, hyperbaric oxygen, and intravitreal injection of corticosteroids or anti-VEGF agents [45].

Maculopathy and optic nerve neuropathy occur in 25% and 8–14% of patients, respectively, after RT [46,47]. These complications severely affect visual acuity [48]. The most important risk factors for their development are tumor location, thickness, volume, and total dose given to the fovea [49,50]. Radiation-induced maculopathy may be effectively managed by intravitreal injections of anti-VEGF drugs or dexamethasone [51].

A less frequent but severe complication is secondary glaucoma that is typically refractory to intraocular pressure-reducing agents. It occurs in 2–15% of patients who underwent eye RT and is the second most frequent reason for enucleation after irradiation [52,53]. The risk factors for secondary glaucoma are larger and thicker tumors, more advanced age, chronic retinal detachment, and high tumor vascularity [46,52]. The available treatment methods include intravitreal injection of anti-VEGF agents, trans-scleral cyclophotocoagulation, and enucleation [54,55].

In summary, RT is an effective but relatively toxic treatment for uveal melanoma. The proper qualification should include a comprehensive ophthalmologic and oncologic assessment of the risks and benefits of each RT method. The development of equally effective but less toxic eye-sparing treatment strategies is warranted.

## 4. Eye-Preserving Surgical Resection

Another possible treatment of uveal melanoma is surgical resection. Surgical treatment leads to avoiding functional blindness caused by enucleation and allows histopathologic and cytogenic analysis. Surgical procedures involved transretinal (endoresection) and transscleral (exoresection), which are technically difficult and require highly experienced surgeon-ophthalmologist and health professional staff. Surgical eye-preservation techniques can be applied in small melanoma or choroidal naevus. Furthermore, large tumors may be removed by transscleral resection, but eye-retaining treatment may be applied if patients do not qualify for radiotherapy. Local resection could be used when iris and ciliary body melanomas occur [3,56,57]. Compared to (^125^I) brachytherapy, transscleral tumor resection treatment incidence of secondary glaucoma is lower, and patients retain a better visual function [58,59]. Eye-preserving methods enable vision preservation, but it may also be associated with many complications like retinal detachment, ocular hypertension, and submacular hemorrhage. Another disadvantage of these methods is repeated reoperation. Patients must be informed about the risk of treatment like visual loss or metastasis [3,59]. After vitreoretinal surgery, local recurrence risk after five years of treatment is 10.4%, and metastasis occurs at a 40.3% level [60].

## 5. Photodynamic Therapy

Photodynamic therapy (PDT) is a commonly used modality in treating various kinds of eye diseases, including UM [61]. Photodynamic therapy (PDT) action is based on the selective destruction of cancer cells or pathological vessels. The PDT mechanism uses light to activate photosensitizers, generating reactive oxygen species that kill cells [62,63,64]. Due to its specific mechanism of action, photodynamic therapy minimizes damage to normal cells [62]. An important factor determining the effectiveness of this method is the selection of the photosensitizer, which should preferentially accumulate in cancer cells and be susceptible to light activation.

Additionally, it should be non-toxic to normal cells. An example of such a compound is Tanshinone IIA, which accumulates in the nucleus of human choroidal melanoma MUM-2B cells and, upon light activation, generates ROS and induces apoptosis [65]. Another group of compounds commonly used in PDT is porphyrins and their derivatives. Studies carried out by Leviskas et al. have shown that Metalloporphyrin Pd (T4) used alone or in combination with 5-aminolevulinic acid (the porphyrin synthesis precursor) is effective in PDT against highly invasive uveal melanoma cell line C918 in vitro [63].

Currently, in clinical settings, a derivative of porphyrin—verteporfin—is used as a photosensitizer (Supplementary Table S1). Although it is approved for treating AMD and CNV [45], studies have shown that verteporfin-mediated PDT is an effective, safe, and well-tolerated method of uveal melanoma treatment [66,67]. In a case series conducted on 15 patients with small pigmented posterior poles, choroidal melanoma response to treatment was confirmed in 12 patients. The main outcomes were reduced subretinal fluid, improved visual acuity in some patients, and decreased tumor thickness [66,68]. In another study, complete tumor regression was observed in 67% (*n* = 12) and improved visual acuity in one patient and stable results in the others. PDT therapy is also characterized by a good prognosis, allowing patients to maintain good vision. Although numerous advantages were shown in the studies, most observations of patients after PDT were performed for a limited time. Therefore, to confirm the long-term effects of PDT, longer observations are necessary [68,69].

Recent studies carried out by Roelofs et al. indicate a risk of recurrence following PDT, suggesting that PDT with verteporfin should only be applied in these cases of choroidal melanoma, where other treatment methods that could provide better control of the tumor cannot be implemented [70]. A recently published meta-analysis summarized verteporfin-mediated PDT results in uveal melanoma from seven studies involving 162 patients. The main outcomes of this meta-analysis were regression of the disease and response to treatment observed in 80% of patients, with a mean follow-up of 50 months [71]. It is worth considering improving PDT or finding a more effective method with a similar mechanism. Perhaps sonodynamic therapy could be a better therapeutic option. It works similarly to PDT, with the difference that ultrasounds are used to activate the photosensitizer. The potential advantages and possibilities of using SDT in ocular oncology are described in the later section.

## 6. High Intensity Focused Ultrasound Ablative Technology

In recent years, an innovative therapeutic approach using High-Intensity Focused Ultrasound (HIFU) has been proposed to treat solid tumors located in various organs. The HIFU technique is a promising and dynamically developing technology of thermal destruction of solid tumors located deep under the skin due to its non-invasive nature (without surgical intervention), lack of ionization, the possibility of repeated treatment, minimal pain for the patient, low treatment and operating costs, as well as minimal side effects. In clinical practice, the ablative technique using HIFU has been used recently to treat solid tumors of the prostate [72], liver [73,74], kidney [73], or breast [73,75,76,77,78] cancers, as well as uterine fibroids [79].

**Figure 3:**
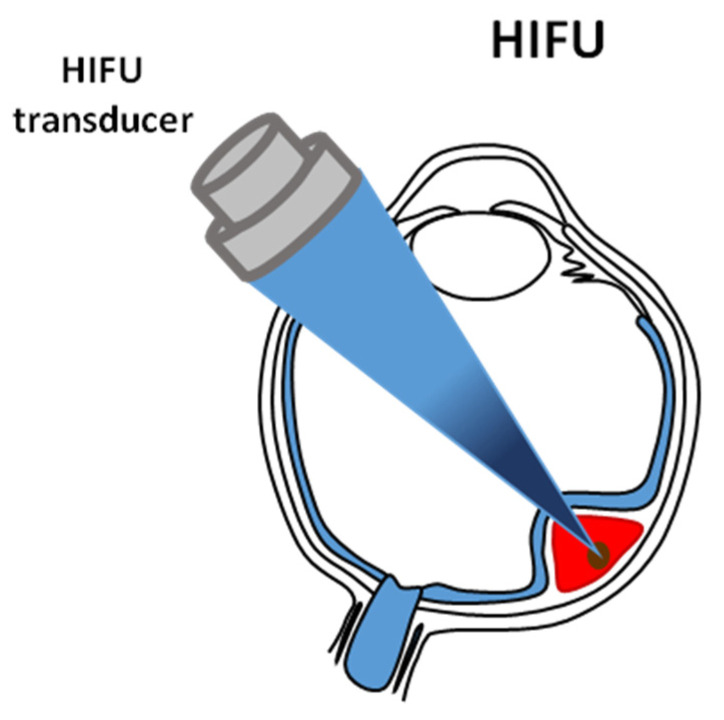
An illustration of High Intensity Focused Ultrasound ablative technology. This technique is based on very quick heating of a small local volume inside the treated tumor, leading to its coagulation necrosis and cavitation.

This technique is based on a very quick (<3 s) heating of a small local volume inside the treated tumor to a temperature above 56 °C, leading to its coagulation necrosis [80] due to the absorption of the energy of ultrasonic waves concentrated in the focal volume of the beam (Figure 3), as well as due to the cavitation [81] that destroys the tissue mechanically. The critical condition is to raise the temperature very quickly to a cytotoxic level so that the tissue vascular system does not significantly influence the volume of damaged tissue. The extent of the ellipsoidal volume of the necrotic lesion formed by the HIFU beam reflects its focal volume. Its size depends on the geometry and acoustic properties of the HIFU beam used and the acoustic and thermal properties of the tissues through which the ultrasonic waves propagate.

The typical dimensions of the ellipsoidal necrotic lesions are as follows: the diameter is in the order of one acoustic wavelength λ, and the length is in the order of 5–7 λ [82]. For example, for a 1MHz HIFU beam, the wavelength in soft tissues is approximately 1.6 mm. Therefore, the necrotic lesion created by such a beam will have approx. 1.6 mm in diameter and approx. 10 mm in length. Meanwhile, for a beam with a frequency of 10 MHz, the diameter of the necrotic lesion will be about 0.16 mm, and the length about 1 mm. The microscopic image of necrosis induced by HIFU differs from that caused by ischemia. The margin between completely damaged cells and healthy tissues is not more than 50 µm [83]. High precision of the therapy is very important for the patient’s safety.

To cover the entire tumor with necrosis, it is necessary to penetrate its entire volume with the focal volume of the HIFU beam. The entire tumor volume can be ablated by scanning it with a series of single exposures to the HIFU beam moved along a programmed trajectory (with a selected distance and time interval between exposures) using a mechanical precision positioning using an electronic control system [80]. 

The concentration of energy of ultrasonic waves into a small local volume inside the tumor can be achieved by using both single-element piezoceramic HIFU transducers in the shape of a spherical bowl of large diameter and by arranging many small piezoceramic transducers on the surface of the spherical bowl. Depending on the excitation mode, the HIFU transducer can generate continuous or pulsed waves. In older generation devices, the tumor is scanned using a HIFU beam generated by a single-element transducer with a fixed focal length, moved in 3D space by a mechanical precision positioning system [84]. Thanks to the development in the field of electronics and the technology of producing multi-element piezocomposite transducers, it has become possible to build new generation HIFU devices in which the focusing of the beam in 3D space and time, as well as the scanning of the tumor by this beam, are carried out using electronic control of the amplitude and time delay of pulses exciting each element of the transducer separately [80].

Multi-element phased-array HIFU probes provide faster movement of the HIFU beam focus within the tumor and greater possibilities of adjusting its geometric dimensions thanks to the flexibility of electronic control and the ability to create multiple foci at once, the spatial synthesis of which leads to a shorter treatment time. Since the heterogeneity of the tissues through which the pulsed focused ultrasound waves propagate can reduce the focus sharpness, especially in tumors located deep under the skin, various methods of phase correction are used to ensure the safety of the therapy for the patient [80].

The choice of the optimal frequency of the HIFU beam depends on the organ to be treated and is a compromise between the depth of the tumor under the skin and the desired rate of temperature increase. The more shallowly the tumor is located under the skin, the higher the HIFU beam frequencies used. For example, to treat tumors located inside the eye (e.g., uveal melanoma), a HIFU beam with a frequency above 10 MHz would be needed [80,84]. 

HIFU transducers in ablative devices have a large radiation aperture, and the ratio of their diameter to the radius of curvature is smaller or close to 1. The choice of such transducers is dictated by the beam they generate, which should have a large opening angle. As a result, it penetrates deep into the body by passing through a large skin surface, where its intensity is much lower than at the focus of the HIFU beam. This helps to avoid skin burns [80].

To couple the acoustic impedances of the HIFU transducer and tissue, a matching medium is used, usually water, which is also a cooling medium. Planning the therapy and monitoring and controlling its course may be achieved using two visualization techniques: ultrasound imaging (USI) or magnetic resonance imaging (MRI).

So far, the main clinical application of the HIFU technique in ophthalmology is the treatment of patients with refractory glaucoma. For this purpose, older-generation HIFU devices have been used. Devices such as the Sonocare CST-100 (SonocareInc, Ridgewood, NJ, USA) have been used in clinical practice primarily for the treatment of eyes with refractory glaucoma by thermal damage to the ciliary body (HIFU cyclocoagulation), leading to a significant reduction in intraocular pressure [85,86]. However, due to the complexity and duration (approx. 2 h) of the treatment procedure, as well as the relatively large focal volume of the HIFU beam used, resulting from its too low frequency (5 MHz) and leading to complications caused by damage to adjacent healthy tissues, the use of this device was discontinued. In 2011, to reduce intraocular pressure in patients with refractory glaucoma, Aptel et al. used circular ultrasound cycloagulation using HIFU beams generated by six rectangular concave transducers evenly spaced on the surface of the annular segment of the sphere [87]. Six locations around the circumference, 1 mm behind the corneal limbus, were subjected to continuous ultrasound waves focused on the ciliary body, causing thermal damage to the body at six locations. For this purpose, a miniaturized HIFU EyeOP1 device was built. 

The use of high-intensity focused ultrasound in oncology has been studied for many years. However, the standards for using the HIFU technique for the ablative treatment of uveal melanoma are much stricter than any other anatomical organ. Such requirements would be met by new-generation miniaturized multi-element phased array HIFU devices. The position and size of the focal volume of the HIFU beam can be electronically controlled, synthesized, and guided to the treated tumor volume using magnetic resonance imaging combined with thermometry or utilizing high-frequency ultrasound imaging.

At the current stage of development, such devices have not yet been created. However, the implementation of miniaturized multi-element high-frequency (>10 MHz) HIFU phased array transducers capable of generating pulsed beams with electronically steered and synthesized focus, targeted on the treated tumor using MR imaging combined with thermometry or high-frequency ultrasound imaging will open up new perspectives for development HIFU techniques in the treatment of various eye diseases including uveal melanoma. Such a new generation device that will ensure the effectiveness and safety of therapy has a good chance of achieving commercial success. 

## 7. Sonodynamic Therapy

**Figure 4:**
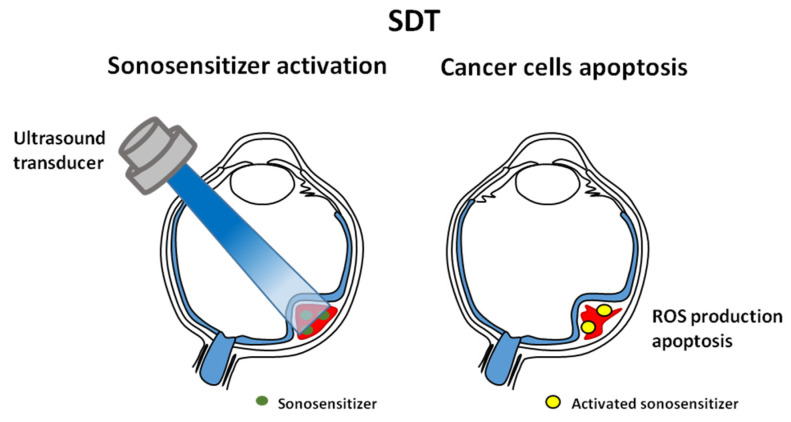
An illustration of sonodynamic therapy. This technology was developed from photodynamic therapy. Both induce reactive oxygen species (ROS) and kill cancer cells. The effect is mediated by the photosensitizers.

Sonodynamic therapy (SDT) was developed from photodynamic therapy (PDT) [1]. A similar effect of both therapies is to induce the reactive oxygen species (ROS) and kill cancer cells, but the excitation mechanisms of SDT and PDT are different (Figure 4). Moreover, SDT relies on the synergy of ROS production and mechanical pressure. It damages the mitochondrial membrane (and structures on its surface) opposite PDT, where this membrane remains intact. SDT is less invasive than PDT—it does not require endoscopic pierced optical fiber or surgical exposure of the tumor, guidance by CT or MRI. Ultrasounds may penetrate deep into tissues (Figure 3), in contrast to photodynamic treatment in which light permeability to deep tumor tissues is limited [88,89]. 

Treated lesions are accessible, and this type of therapy is less effective for large tumors. Usage of photosensitizers is associated with the necessity of avoiding sunlight for several weeks by a treated patient. Moreover, SDT kills cancer cells by simultaneously reducing the damage of adjacent normal tissue [90]. SDT gives a better outcome on high pigmentation melanoma skin cancer than PDT [91,92].

The detailed mechanism of SDT is still unclear. Sound waves deliver a portion of energy absorbed by the sonosensitizer, and its excited electrons initiate chemical reactions with biomolecules and water. The products of these reactions are free radicals. Consequently, sonodynamic therapy induces a cavitation effect, generation of free radicals, and direct apoptosis of cancer cells. Cavitation is divided into non-inertial and inertial. Non-inertial cavitation occurs in low-intensity ultrasound. Cavitation bubbles that appear in water oscillate and affect surrounding suspended particles. Inertial cavitation occurs when liquid is subjected to high-intensity ultrasound. Bubbles absorb more energy and release it on a small area, resulting in high temperature, pressure, and generation of free radicals [91]. The biological effect on cells of non–inertial cavitation is limited to changing membrane permeability. Inertial cavitation may destroy the cytoskeleton, cell membrane structures, and enzymes, killing surrounding cells [91]. Tumor treatment requires both types of cavitation (damaging tumor cells by protecting the surrounding tissues or destroying tumor cells with some margin). Non-inertial cavitation may turn into inertial [91].

Sonodynamic therapy induces apoptosis. A large quantity of ROS produced during SDT reduces mitochondrial membrane potential and leads to apoptosis. SDT also induces the expression of Bcl-2 family proteins, increases the amount of BAX/BAX or BAX/Bcl-2 dimers, which leads to apoptosis. STD leads to Ca^2+^ overload. In vitro studies on glioma cell line (C6) show that treating cells with low-level ultrasound in combination with hematoporphyrin monomethyl ether (HMME) increases ROS production and the intercellular Ca^2+^ level, decreases mitochondrial membrane potential, and releases cytochrome c [93]. HMME-SDT or protoporphyrin IX (PpIX)-mediated sonodynamic therapy (PpIX-SDT) also induces apoptosis in leukemia U937 and K562 cell lines. Changes after sonodynamic treatment on the cellular level include nuclear morphology (chromatin condensation), translocation of BAX protein (from the cytoplasm to mitochondria), and caspase activation [94,95]. Apoptosis activation by SDT may also occur by activating exogenous pathways and up-regulating the expression of FAS/FASL receptors [91].

Low-intensity therapeutic ultrasound (5-aminolevulinic acid as a sonosensitizer) in mice transplanted with B16F10 melanomas activates a local immune response. M1 type macrophage cells, a high level of inflammatory cytokines TNF-α and IFN-γ, and faster maturation of dendritic cells were observed in the tumor microenvironment [95].

Some sonosensitizers are derived from photosensitizers, e.g., protoporphyrin derivatives [90]. First, SDT was applied to treat mouse sarcoma and rat ascites hepatoma cells by hematoporphyrin in the acoustic field [96]. This group of compounds consists of hematoporphyrin (Hp), photofrin, hematoporphyrin monomethyl ether (HMME), protoporphyrin IX (PpIX), ATX-70, and their novel derivatives. These compounds were tested in cell and animal models in several tumor types (mammary or breast cancer, glioma, osteosarcoma, or leukemia) [90].

A newer group of compounds used in SDT therapy are nano-sensitizers. These compounds possess good solubility and could prolong blood circulation and accumulate in tumor lesion sites [97]. They can be divided into intrinsic sonosensitizers (titanium dioxide—TiO_2_; nanoparticles—NPs) and nanoparticle-assisted sonosensitizers. From the first group, the most extensively studied is TiO_2_. It is low-cost and easy to produce, and due to its semiconductor properties, it may generate ROS [98]. In melanoma cell line (C32), irradiation by ultrasound in the presence of TiO_2_ results in damage of cell membranes and induction of apoptosis. In the mouse in vivo model, a combination of SDT with TiO_2_ resulted in significant inhibition of tumor growth compared with untreated mice. Histopathological analysis of tumors shows the presence of necrotic cells and neutrophils [99].

Another group of sonosensitizers are xanthenes. Compounds belonging to this group like eosin, fluorescein, and rhodamines possess good water solubility [90]. One of them is Rose Bengal, which is a fluorescein derivative. It is used to stain the ocular surface epithelium to assess damage in ocular surface diseases [100]. It was tested in melanoma cell lines [101,102]. In A375 cells, after sunlight exposure, it causes a phototoxic effect resulting in DNA damage and apoptosis of tumor cells. It is recommended to avoid natural sunlight exposure after using Rose Bengal [102].

Attempts to apply sonodynamic therapy have been made concerning many types of neoplasms, such as glioblastoma, lung adenocarcinoma, human lung adenocarcinoma, leukemia, melanoma, sarcoma, squamous tongue carcinoma, breast and hepatocellular carcinoma [103]. So far, this form of therapy has not been studied for uveal melanoma. SDT seems to be safer and more specific than PDT. Ultrasound can be tightly focused with good penetration through soft tissue [104]. There is a possibility of non-invasive ultrasound of the eye (through the front of the eye). Still, the application of several transducers should be tested in vivo for their safety and effectiveness.

## 8. Electrically Enhanced Drug Delivery

**Figure 5:**
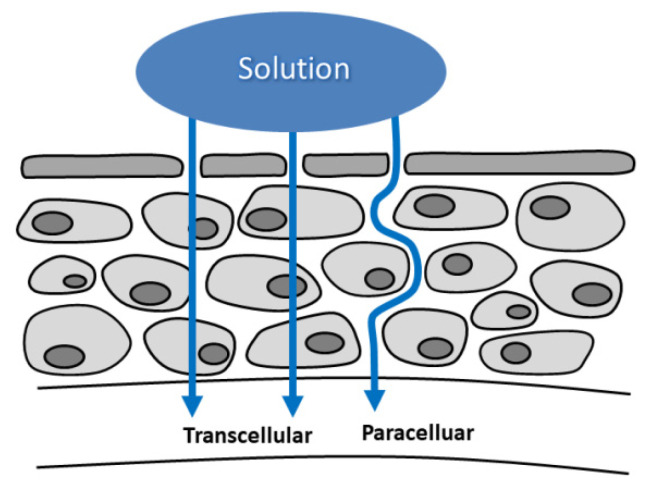
An illustration of electrically enhanced drug delivery. The applied drug may be transported either paracellularly or transcellularly.

Enhancing drug delivery with physical forces and specifically with the application of the electric current (Figure 5) seems to be a new promising approach in many oncological applications [105]. In the case of ocular oncology, and specifically UM, two approaches could be currently considered: iontophoresis and electrochemotherapy (ECT).

Iontophoresis is a non-invasive technique in which using low-intensity electric current allows to increase the biodistribution of ionized drug molecules in the tissues of the eyeball, particularly in the cornea and sclera. Transcorneal iontophoresis has been shown to increase the local concentration of antibacterial and antifungal drugs, steroids, DNA, and RNA molecules [106,107]. EyeGate company has developed a transscleral iontophoresis device and has completed Phase III clinical trials of EPG-437 formulation (dexamethasone formulation developed for iontophoresis administration) for anterior uveitis. The treatment results were similar to the standard therapy (prednisolone in the form of eye drops). Still, the risk of increased intraocular pressure and the frequency of drug administration was lowered [108].

Iontophoresis with carboplatin delivery was a promising option for retinoblastoma treatment. Transcorneoscleral delivery of the drug results in dose-dependent inhibition of intraocular retinoblastoma. In the mouse model, a 7.0 mg/mL dose was evaluated as a tumor control dose for 50% of treated eyes. At this dose, no corneal toxicity signs were observed [109]. There were no toxicity signs in the rabbit eye (more anatomically similar to the human eye) after six transscleral applications of carboplatin at 14 mg/mL [110].

The disadvantage of the iontophoresis technique is its low effectiveness to deeper tissues of the eyeball, its limited effectiveness of supporting the internalization of drug molecules into cells, and its limited ability to precisely administer drugs locally. However, there are attempts to deliver nanoparticles to sites near the posterior pole region of the eye. In ex vivo and in vivo animal models, iontophoresis using microneedle-based devices allows delivering charged nanoparticles to the posterior region of the suprachoroidal space (SCS; >9 mm from the limbus) with average 6 nm penetration [111].

In electrochemotherapy, electric pulses generate and open transient pores in the cell membrane and enable the influx of drug (chemotherapeutic) molecules into the cytosol. Its principal advantage is local dose intensity. A high intratumoral drug concentration is achieved, and cytotoxicity is increased by ~8000 fold for bleomycin and ~80 fold for cisplatin [112]. Besides electropermeabilization and electrophoresis, other mechanisms that might also play a role in electroporation are passive diffusion, convection, macropinocytosis, and endocytosis (electroendocytosis) possible uptake-mechanisms for neutral particles during electroporation. Recently, a meta-analysis of ECT clinical trials showed that the overall objective response rate (ORR) ranges from 62.6% to 82.2%, depending on the drug type and route of administration, type of cancer, and tumor size. Over the last few years, great efforts have been made to extend ECT to non-cutaneous tumor locations, including the liver, pancreas, bones, and brain. Moreover, an endoscopic electrode was developed to treat colon and rectal cancer [113].

In preclinical studies, several centers use needle-like electrodes for localized electroporation in the postnatal brain in rats and mice. Electroporation does not result in behavioral side effects, and no motor response or seizure-like activity was observed. Most recently, in vivo single-cell electroporation was used in rats for Ca^2+^ indicator loading. Successful loading of these tracers into the neurons was also confirmed [114]. The extracellular matrix composition influences electroporation efficiency: soft tumors with larger spherical cells, low proteoglycan and collagen content, and low cell density are more effectively transfected [115]; therefore, uveal melanoma seems like a good potential candidate for that type of treatment.

In the case of intraocular administration, an additional limitation is the presence of the blood–retinal, blood–aqueous humor, and blood–vitreous barriers. It seems that electroporation should bring the greatest benefits in the case of administering drugs with low permeability through biological, hydrophobic barriers. ECT was evaluated in in vitro (spheroid) and in vivo (chick embryo chorioallantoic membrane, CAM) primary and metastatic UM models. Compared to chemotherapy or electroporation, ECT caused a reduction in tumor size and viability of tumor cells. Spheroids treated by ECT (with bleomycin in lower concentration 2.5 µg/mL than peak plasma) lose sphericity. Peripherally located cells detach from the main spheroid body. ECT treatment changes the spheroid shape, alters the inner area core with necrotic cells, and the outer area consists of proliferative cells [116,117]. Bleomycin combined with electroporation reduced the viability of conjunctival cell lines (CRMM1, CRMM2). Electroporation highly enhances the activity of bleomycin chemotherapy in vitro [118]. In the in vivo model, apoptosis and necrosis areas in the peripheral graft region were observed after intraarterial infusion in the tumor’s proximity. The intratumoral treatment gives large necrosis in the center of the tumor mass [117].

Simulation studies on the 3D mathematical model of the eye show that nonthermal irreversible electroporation can be safely applied to treat intraocular tumors [119]. Ex vivo experiments demonstrated ablation of uveal melanoma tumors, but tumor conductivity increased during treatment [120]. Optimization of pulse parameters and electrode configuration are important factors before planning treatment. Animal and human studies are still needed to develop ECT for clinical use [119].

## 9. Theranostics

**Figure 6:**
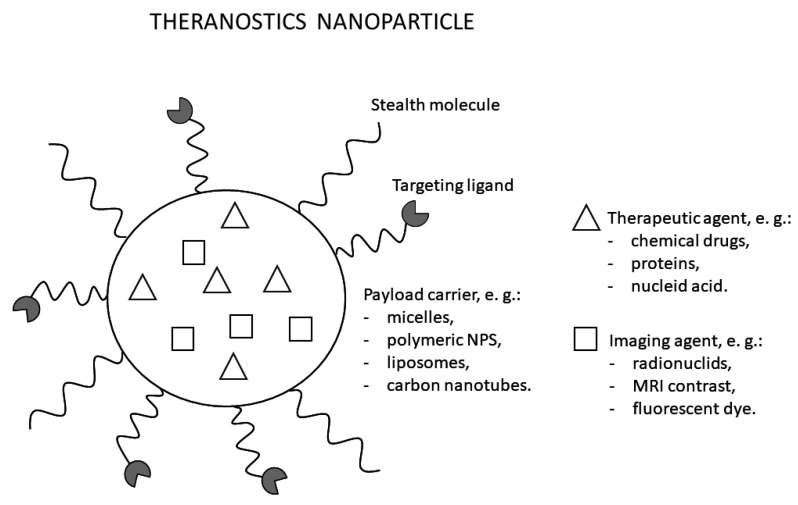
An illustration of a nanoparticle used for theranostics. The particle enables imaging, cancer-targeting, and therapeutic effect.

Theranostics, also known as theragnosis, is a modern technique in personalized medicine incorporating both diagnostic imaging and therapy. Instead of utilizing two different materials for both purposes, theranostics uses a single probe combing two features into one platform (Figure 6) [121]. This dual property allows recognition of the specific disease, understanding the cellular phenotype, and provides immediately targeted treatment to monitor and observe its efficacy [122,123]. Therefore, most direct targets in oncology of this method include antigens and receptors expressed specifically by certain tumor cells (e.g., insulin-like growth factor 1 receptor—IGF1R; epidermal growth factor receptor—EGFR; human epidermal growth factor receptor 2—HER2), elements of the tumor microenvironment as well as altered cell metabolism or hypoxia, and extracellular acidosis caused by abnormal vasculature. Such cancer-specific targeting results in selective action of anti-cancer substances and limiting or even eliminating systemic side effects by reducing its harmful effects on healthy tissues [124]. 

Nuclear oncology is one of the main fields which integrated theranostics into clinical practice. Radiopharmaceuticals with γ-emitting or positron-emitting radionuclides can be easily visualized by positron emission tomography (using emitters such as fluorine-18 or gallium-68), or single-photon emission computed tomography (with the use of emitters such as technetium-99m). When labeled with β-emitting radionuclides (e.g., lutetium-177), they can be utilized as a treatment modality [122,125].

One of the most remarkable achievements of the modern theranostic radionuclide approach was accomplished with the NETTER-1 study [126]. After many years of clinical development, 177-lutetium-DOTA-octreotate for peptide receptor radionuclide therapy (PRRT) of gastroenteropancreatic neuroendocrine tumors (NETs) obtained overall approval. A landmark study involving patients with advanced somatostatin-receptor positive mid-gut NETs was published in 2017. It led to the elevation of PRRT to level 1b evidence and FDA approval of 177-lutetium-DOTA-octreotate PRRT of gastroenteropancreatic NETs [127,128]. Additionally, currently, a new somatostatin-receptor antagonist (the pair 68-gallium-JR11 and 177-lutetium-DOTA-JR11) is tested in patients with not only gastroenteropancreatic NETs but also other cancers, including bronchial carcinoid or phaeochromocytoma (NCT02592707) [127]. Current receptor ligands might be labeled with new radionuclides, e.g., 47-scandium, 161-terbium, 213-bismuth, as evaluated in preclinical trials [128].

Theranostics is a field that strongly benefits from the fast development of nanomedicine. Nanoparticles (NPs) offer multifunctionality as they can integrate a few imaging or therapeutic agents and enhance circulation time in the blood. Additionally, by controlling the size and shape of NPs, different demands of biological systems can be met [129,130]. Theranostics NPs are engineered in several ways, e.g., by encapsulating therapeutic and imaging agents in platforms such as micelles and polymeric NPs, or by loading therapeutic agents into existing imaging NPs such as gold nanocages and iron oxide NPs, or quantum dots. Theranostics NPs surface is also modified with specific targeting ligands and polyethylene glycol to allow active tumor targeting and improve the blood circulation half-life [131]. However, many limitations still exist, including costs and toxicity (e.g., impairment of mitochondrial function, DNA damage) that must be studied and evaluated before introducing theranostics NPs in everyday clinical practice [121,132].

One of the first introduced theranostics NP was Herceptin^®^, developed to treat metastatic breast cancer with HER-2 overexpression [121]. Since then, a lot of new developments have arrived. NPs with anti-cancer drugs (e.g., Doxorubicin or Paclitaxel) were combined with imaging agents for simultaneous imaging and targeted chemotherapy. Kim et al. [133] introduced chitosan-based NPs labeled with Cy5.5 (fluorescent dye) for imaging purposes and loaded them with an anti-cancer drug, paclitaxel. The compound exhibited high accumulation in tumor tissues and resulted in high therapeutic efficacy. Other examples include EGFR-targeted liposomes loaded with DNA bio-dots and a combination of anti-cancer drugs cetuximab and etoposide in treating advanced non-small cell lung carcinoma [134].

Additionally, in the treatment of glioblastoma, circulating tumor DNA is evaluated as a theranostic marker (NCT03115138). New compounds and materials with appropriate modification and use of long-known anti-cancer substances allow for selective cancer management. 

Although no theranostic NPs have been developed specifically for uveal melanoma, some NPs have already found use in ophthalmology. With limitations from conventional therapies during drug delivery to the eye, nanotechnology-based drug delivery allows better uptake across ocular barriers, sustained drug release, and tissue targeting. For example, Restasis^®^ was developed as a nanoemulsion with cyclosporin A to treat chronic dry eye. At the same time, intravitreal injection with Macugen^®^, an anti-vascular endothelial growth factor, was approved for age-related macular degeneration treatment [135,136]. With increased bioavailability and drug targeting, theranostic NPs for use in imaging and treating uveal melanoma could be an attractive modality, especially in patients with early-stage disease, to reduce side effects and spare healthy tissues of the eye.

## 10. Conclusions

Uveal melanoma is the most common intraocular malignancy and arises from melanocytes in the choroid, ciliary body, or iris. UM signs and symptoms are non-specific and include high intraocular pressure, myodesopsia, photopsia, and, finally, loss of vision. The management of localized UM is either globe-preserving therapy or enucleation. Globe-preserving therapies may be surgical, radiation therapy, or laser therapy.

Ocular pharmacology is unique. The drug administration routes and therapeutic challenges vary depending on the eye segment. The anterior segment of the eye includes the cornea, iris, ciliary body, and lens. Topical application is the most common form of pharmacotherapy in this segment.

Radiotherapy is the mainstay of therapy for most patients with localized uveal melanoma. Due to the predicted radioresistance of melanoma cells, high fraction doses are required to achieve satisfactory response and local control. Another RT technique used in the treatment of uveal melanomas is charged-particle radiotherapy. It includes protons, carbon ions, and helium ions. Unfortunately, the availability of CRT, especially ion therapy, remains poor due to the high cost of equipment and treatment.

Among eye-preserving methods in treatment of UM, surgical resection is one possible treatment option. Despite it being a clinical challenge and associated with many complications, it allows vision preservation and retains a better visual function vs. brachytherapy. Photodynamic therapy is a commonly used modality in treating various kinds of eye diseases, including uveal melanoma. Photodynamic therapy action is based on the selective destruction of cancer cells or pathological vessels. The PDT mechanism uses light (laser energy) to activate photosensitizers, generating reactive oxygen species that kill cells. Additionally, it should be non-toxic to normal cells. Currently, in clinical settings, verteporfin is used as a photosensitizer. Although it is approved for the treatment of AMD and CNV, studies have shown that verteporfin-mediated PDT is an effective, safe, and well-tolerated method of uveal melanoma treatment.

High-Intensity Focused Ultrasound has been proposed to treat solid tumors located in various organs. The HIFU technique is a promising and dynamically developing technology of thermal destruction of solid tumors located deep under the skin. This technique is based on very quick heating of a small local volume inside the treated tumor to a temperature above 56 °C, leading to its coagulation necrosis due to the absorption of the energy of ultrasonic waves concentrated in the focal volume of the beam, as well as due to the cavitation that destroys the tissue mechanically. The critical condition is to raise the temperature very quickly to a cytotoxic level so that the tissue vascular system does not significantly influence the volume of damaged tissue.

Sonodynamic therapy was developed from photodynamic therapy. A similar effect of both therapies is to induce the reactive oxygen species and kill cancer cells, but the excitation mechanisms of SDT and PDT are different. SDT is less invasive than PDT. Ultrasounds may penetrate deep into tissues, in contrast to photodynamic treatment in which light permeability to deep tumor tissues is limited.

Electroporation applied in vivo delivers drugs or genetic material from the intercellular space to the cells by temporarily permeabilizing cell membranes using a short-term high voltage electrical pulse. The first stage is the introduction of a substance (drug, DNA) into the intercellular space. This approach can be achieved through intravenous or local administration (e.g., intratumorally, directly into cancerous tissue). Intravenous administration is less effective in tumors because of the usually increased pressure in the intercellular space. Data from the in vitro studies indicate that electroporation-assisted administration of chemotherapeutic agents in ocular neoplasms may be a promising new therapy.

Iontophoresis is a non-invasive technique in which using a low-intensity electric current allows to increase the biodistribution of ionized drug molecules in the eyeball tissues, particularly in the cornea and sclera. Transcorneal iontophoresis has been shown to increase the local concentration of antibacterial and antifungal drugs, steroids, DNA, and RNA molecules.

Theranostics is a modern technique in personalized medicine incorporating both diagnostic imaging and therapy. Instead of utilizing two different materials for both purposes, theranostics uses a single probe combing two features into one platform. Cancer-specific targeting results in selective action of anti-cancer substances and limiting or even eliminating systemic side effects by reducing its harmful effects on healthy tissues. Although no theranostic markers have been developed specifically for uveal melanoma, some NPs have already found use in ophthalmology. With limitations from conventional therapies during drug delivery to the eye, nanotechnology-based drug delivery allows better uptake across ocular barriers, sustained drug release, and tissue targeting.

Definitely, treatment for uveal melanoma presents an unmet clinical need. More novel eye-preserving therapeutic approaches for localized disease are desperately needed. Both preclinical research and clinical trials would help to develop these therapies.

## Figures and Tables

**Table 1:** Selected characteristics of radiotherapy modalities used to treat uveal melanoma.

	Brachytherapy	Particle Therapy	Photon Stereotactic Body Radiotherapy
Availability	Moderate	Low	High
Cost	Moderate	High	Low
Tumor size	Small, medium	Medium, large	Medium, large
Specific toxicity	Visual acuity loss, immediate procedural discomfort	Anterior eye complications	
Indications	Majority of uveal melanomas (also with limited extrascleral extension)	Tumors surrounding the optic disk and fovea; an attempt of eye-sparing treatment in large tumors	Rapidly growing tumors
Particular contraindications	Gross orbital extension, blind painful eyes, no light perception	None	Young age predicted long survival (higher late complications rate)

## Supplementary Materials

The following supporting information can be downloaded at: https://www.mdpi.com/article/10.3390/cancers14010134/s1, Table S1: Ongoing clinical trials investigating new treatment modalities for localized uveal melanoma.
