# Supplementary material for: New Perspectives for Eye-Sparing Treatment Strategies in Primary Uveal Melanoma
Source: Cancers (Basel). 2021 Dec 28;14(1):134. doi: 10.3390/cancers14010134 (PMC8750035; doi:10.3390/cancers14010134)
Supplement: Supplementary file 1 [file cancers-14-00134-s001.zip › cancers-1470933 - sup.pdf]

Table S1. Ongoing clinical trials investigating new treatment modalities for localized uveal melanoma. Clinical trials were searched on https://www.clinicaltrials.gov and https://www.clinicaltrialsregister.eu with terms "uveal melanoma" and "uveal melanoma treatment" on 10/15/2021.

Investigated treatment	Clinical trial	Country	Phase
Autologous dendritic cells loaded with autologous tumor RNA	NCT01983748	Germany	Phase 3
Selumetinib-methyl ethyl ketone (MEK) inhibitor in patients with unresectable uveal melanoma	NCT02768766	USA	Phase 1
Pegylated arginine deiminase (ADI-PEG 20), combined with immunotherapy drugs nivolumab and ipilimumab in patients with unresectable uveal melanoma	NCT03922880	USA	Phase 1
Hypofractionated linear accelerator radiotherapy	NCT00872391	Austria	Not-applicable
Endoresection or transpupillary thermotherapy when endoresection is not feasible in patients with large uveal melanoma	NCT02874040	France	Not-applicable
Intravitreal injection of ICON-1 (human immuno-conjugate 1)	NCT02771340	USA	Phase 1
Stereotactic radiotherapy followed by intravitreal aflibercept injection	NCT03712904	USA	Phase 2
Interferon alfa-2b with dacarbazine in patients with proven monosomy 3 and/or 8q amplification	NCT01100528	USA	Phase 2
Suprachoroidal administration of AU-011 (human papillomavirus virus-like particles conjugated with IR700 dye AU-011) in patients with small choroidal melanoma	NCT04417530	USA	Phase 2
Light-activated AU-011 in patients with small choroidal melanoma	NCT03052127	USA	Phase 1B/2
Ziv-aflibercept (VEGF blocker) in patients with inoperable uveal melanoma	NCT00450255	USA	Phase 2

Autologous dendritic cells loaded with autologous tumor RNA in patients with proven monosomy 3 after surgical resection of uveal melanoma to prolong disease-free survival	2007-007847-28	Germany	Phase 3
